# Association of long-term exposure to air pollutants with benign prostatic hyperplasia among middle-aged and older men in China

**DOI:** 10.1007/s00420-025-02127-w

**Published:** 2025-03-14

**Authors:** Wenming Shi, Jie V. Zhao

**Affiliations:** School of Public Health, Li Ka Shing Faculty of Medicine, The University of Hong Kong, Patrick Manson Building, 7 Sassoon Road Hong Kong SAR, Hong Kong SAR, China

**Keywords:** Air pollution, Benign prostatic hyperplasia, Coarse particle, Middle-aged and older adults, PM_2.5_

## Abstract

**Purpose:**

Air pollution has been an important risk factor for human health. However, little is known about the impacts of air pollutants on benign prostatic hyperplasia (BPH) in men. We aimed to explore the association of long-term exposure to air pollutants with BPH among men.

**Methods:**

We leveraged the nationally representative data from the China Health and Retirement Longitudinal Study, a total of 8,826 participants aged 45 years and above from 125 Chinese cities were enrolled in 2015. Annual fine particulate matter (PM_2.5_), coarse particles (PM_2.5−10_), nitrogen dioxide (NO_2_), sulfur dioxide, carbon monoxide, and ozone were estimated using satellite-based models. Multivariate logistic regression models were used to assess the risk of BPH associated with air pollutants. The restricted cubic spline model was performed to explore the exposure-response relationships with BPH.

**Results:**

Of the 8,826 participants (mean age: 60.3 years), the prevalence of BPH was 14.5%. Each 10 µg/m^3^ rise in PM_2.5_ (odds ratio 1.04, 95% confidence intervals: 1.01–1.07) and PM_2.5−10_ (1.06, 1.02–1.10) were associated with prevalent BPH. Compared with the lowest quartile levels, higher PM_2.5_ and PM_2.5−10_ exposure were related to an increased risk of BPH. There were non-linear relationship between PM_2.5−10_ and NO_2_ exposure with prevalent BPH. The association with BPH was more pronounced in participants who were overweight/obesity.

**Conclusion:**

This study suggests that long-term air pollutants exposure, especially for PM_2.5_ and PM_2.5−10_, is associated with BPH among middle-aged and older men. Our findings provide epidemiological evidence for policymakers and researchers to improve prostate health by reducing air pollution.

**Supplementary Information:**

The online version contains supplementary material available at 10.1007/s00420-025-02127-w.

## Introduction

Benign prostatic hyperplasia (BPH), characterized as the unregulated enlargement of the prostate gland, is a common urological disease usually occurring in aging men (Awedew et al. 2022; Zeng et al. 2022). As a main cause of lower urinary tract symptoms (LUTS), BPH followed by substantial LUTS affects 50–75% of men over 50 years (Egan 2016). Previous studies have indicated that BPH is associated with severe morbidity, and complications including urinary tract infection, acute urinary retention, and acute kidney failure (Speakman et al. 2015; Stroup et al. 2012), which brings a heavy burden for patients (Speakman et al. 2015). Consequently, identifying the potential risk factors associated with BPH assumes paramount merits within the dominance of public health.

Air pollution, especially particulate matter (PM), is suggested to be a vital environmental risk of death globally (Tian et al. 2019). Inhalable PM can be divided into fine PM (PM_2.5_) and coarse PM with a fraction size between 2.5 and 10 μm (PM_2.5−10_). Different from PM_2.5_, PM_2.5−10_ is generally formed by mechanical grinding and resuspension of solid substance (Daellenbach et al. 2020), and it typically deposits in the upper respiratory tract or larger airways, and the chemical components with more crustal materials (i.e., silicon, calcium) in PM_2.5−10_ (Peng et al. 2008). The differences in deposition locations and composition suggest that PM_2.5_ and PM_2.5−10_ may have varied effects on people’s health. The potential mechanisms for PM include oxidative stress, DNA damage, systematic inflammation, and endocrine dysfunction (Zhang et al. 2022a). During the past years, a growing number of epidemiological studies have shown that air pollutants are associated with multiple adverse health outcomes including cardiovascular diseases (Kaufman et al. 2016), respiratory diseases (Shi et al. 2021), cancer (Kayyal-Tarabeia et al. 2024), and urinary incontinence symptoms (Liu et al. 2024), and carries a heavy burden. Currently, the etiology of BPH is not well understood, but factors such as hormone disruption, and inflammation are believed to play a role in the progress of this disease (Rastrelli et al. 2019). Existing studies suggest that PM exposure is related to higher testosterone levels in men, and specific PM components, like polycyclic aromatic hydrocarbons (PAH_S_), may possess hormone-modulating properties (Radwan et al. 2016; Wei et al. 2021a, b). However, the potential impact of air pollution on the risk of BPH has been scarcely investigated. To our knowledge, only one study reported that air pollutants exposure, i.e., nitrogen oxides (NO_X_) and PM_10_, was associated with BPH in Korea (Shim et al. 2016), whereas it is based on an ecological study and hence has limited ability of causal inference. There is a notable absence of studies that have assessed the impacts of other air pollutants like PM_2.5_ and PM_2.5−10_ on BPH to date. In addition, whether the demographic and behavior factors modify the associations of air pollutants with BPH is still unknown.

China has a rapidly aging population and is facing several air pollution problems (Liu et al. 2022; PayneXu 2022). To address the research gap, we leveraged the nationally representative data to investigate the association of various air pollutants (PM_2.5_, PM_2.5−10_, nitrogen dioxide (NO_2_), sulfur dioxide (SO_2_), carbon monoxide (CO), and ozone (O_3_) with BPH in China. We also performed an investigation into the potential modification effects of demographic and behaviour factors that may exhibit susceptibility to the association of air pollution with BPH.

## Methods

### Study population

The China Health and Retirement Longitudinal Study (CHARLS) is a nationally representative project among middle-aged and elderly adults in mainland China. The CHARLS aims to collect high-quality microdata among residents aged 45 years or above from 450 villages/communities to analyze the aging in China and improve interdisciplinary studies on aging. Detailed design and sampling approaches have been described previously (Zhao et al. 2014). Initially, 17,708 respondents were recruited from 28 provinces in 2011 using a multistage probability sampling strategy (see *eMethods*), and then were followed up every two to three years. During each survey, the demographic information, lifestyle and behavior characteristics, and health conditions were collected.

In the current study, a total of 21,095 respondents were recruited in the CHARLS 2015. We excluded women, individuals younger than 45 years or without age data, and those with the missing outcome, 8,826 individuals from 125 county-cities (eMethods, Figure S1) across 28 provinces were screened for the final analyses. Figure S2 shows the flowchart of the participants’ selection. The CHARLS was ethnically approved by the Peking University institutional review board (IRB00001052–11015). Informed consent was signed by each participant. Our study was in accordance with the ethical principles of the Declaration of Helsinki.

### Exposure estimation

Data on annual average concentrations of PM_2.5_ and PM_10_ at 1 km resolution (Wei et al. 2021a, b, 2023a, b), NO_2_, SO_2_, CO, and O_3_ at 10 km resolution (Wei et al. 2022, 2023a, b) across China from 2011 to 2015 were retrieved from the ChinaHighAirPollutants (CHAP) dataset (available at https://weijing-rs.github.io/ product.html). The CHAP is a high-quality product for air pollution, which used a conglomeration of multiple sources including ground-based measurements, satellite models, atmospheric reanalysis, and was generated using artificial intelligence to account for the spatiotemporal variations of air pollution. The cross-validation coefficient of determination (R^2^) ranged from 0.80 to 0.92 for the predictions of these six air pollutants (Wei et al. 2021a, b, 2022, 2023a, b). Outdoor PM_2.5−10_ was calculated by subtracting the annual PM_2.5_ from PM_10_. Given the privacy considerations, the specific residential addresses of participants were geocoded at the county-city level in the CHARLS, similar to previous studies (Zhao et al. 2025; Hu et al. 2023; Shi et al. 2023; Han et al. 2022). Exposure to air pollutants was thus evaluated according to the gridded estimates within the 125 Chinese county-cities. We utilized two-year average concentrations of air pollutants before 2015 survey as long-term exposure for each participant in the main analyses.

### Assessment of BPH

In accordance with previous studies from the CHARLS (Xiong et al. 2022; Zhang et al., 2022b), individuals were asked during the face-to-face interview, “Have you ever been diagnosed with prostate hyperplasia (excluding prostatic cancer)? Moreover, researchers also explained the main symptoms of BPH to the participants (Xiong et al. 2022). The diagnosis of BPH was based on a positive response to this question, after understanding the symptoms of BPH.

### Covariates

Demographics included age, body mass index (BMI), education attainment, marital status, and place of residence (urban or rural). lifestyle and Behavior factors including tobacco smoke (never, current, or previous), alcohol use (yes or no, specifying if they had ever consumed alcohol) (Shi et al. 2024), physical activity levels (low, moderate, or high), and the self-rated health status (good, fair, or poor) were recorded. Additionally, annual average temperature and relative humidity (RH) was obtained from the China Meteorological Data Service Center.

### Statistical analyses

The distributions of the baseline characteristics were described, and the χ^2^ test and t-test were adopted for comparison of categorical variables and continuous variables, respectively. The distributions of the exposure levels of air pollutants were described. Spearman’s correlation analyses was conducted to explore the correlation between any two air pollutants.

Multivariable logistic regression was applied to explore the association of the two-year average of air pollutants with prevalent BPH. The estimated odds ratio (OR) was reported for per 10 µg/m^3^ increase in air pollutants except for CO (per 1 µg/m^3^). Both crude and adjusted models were performed to analyze the association. According to the priori knowledge and existing literature (Xiong et al. 2020; Morita et al. 2013; Parsons 2007; Burke et al. 2006), we developed a directed acyclic graph (DAG) to determine which candidate covariate should be adjusted in the multivariate analyses (Figure S3), using the online DAGitty tool (www.dagitty.net). The model finally included age, BMI, educational attainment, marital status, tobacco smoke, alcohol use, self-rated health status, residence, annual temperature, and RH. Moreover, we assessed the relationships by dividing air pollutant levels into four quartiles and calculated the estimates using the lowest quartile as the reference. Trend analyses were conducted by modeling each quartile level of pollutants as an ordinal variable. Furthermore, to examine the exposure-response relationships between air pollutants with BPH, a restricted cubic spline (RCS) model with three knots was conducted.

We performed several stratification analyses by following variables: age (≥ 65 years or < 65 years), overweight/obesity (yes or no; specifying by BMI ≥ 24.0 kg/m^2^), smoke exposure (yes or no), alcohol use (yes or no), and place of residence (urban or rural). Sensitivity analyses by conducting a two-pollutant model to evaluate the association. We examined the robustness of the association by using five-year exposure before the survey as a sensitivity analysis. Considering that only a subset of the respondents were collected data on physical activity in the CHARLS (Li et al. 2020), we adjusted for physical activity in the sub-sample (*n* = 4,398).

All analyses were conducted using STATA software (version 16.0), and two-tailed *P* < 0.05 were considered statistically significant.

### Results

Of the 8,826 male adults (mean age 60.3 [SD 9.8] years), 1283(14.5%) had BPH in 2015. Table 1 shows that individuals with BPH are more likely to be older, unmarried, have a higher BMI, smoke, and consume alcohol, compared with their counterparts.

**Table 1:** The characteristics of the study participants

Characteristics	Total (*N* = 8826)	BPH		*P*-value
		Yes (*N* = 1283)	No (*N* = 7543)	
Age (years)	60.3 ± 9.8	64.0 ± 10.0	59.7 ± 9.6	< 0.001
BMI (kg/m^2^ )	23.41 ± 3.48	23.97 ± 3.57	23.31 ± 3.45	0.001
Education attainment ^#^				
≤ Primary school	4568 (51.8)	623 (48.6)	3945 (52.3)	< 0.001
Middle school	1899 (21.5)	293 (22.8)	1806 (23.9)	
≥ High school	1276 (14.5)	265 (20.7)	1011 (13.4)	
Marital status				
Married	8004 (90.7)	1141 (88.9)	6863 (91.0)	0.019
Unmarried/ widowed	822 (9.3)	142 (11.1)	680 (9.0)	
Residence				
Urban	3440 (39.0)	618 (48.2)	2822 (37.4)	< 0.001
Rural	5386 (61.0)	665 (51.8)	4721 (62.6)	
Smoke status				
Never	2379 (27.0)	334 (26.0)	2045 (27.1)	< 0.001
Current	4481 (50.8)	534 (41.6)	3947 (52.3)	
Previous	1966 (22.3)	415 (32.3)	1551 (20.6)	
Alcohol drink				
Have	5668 (64.2)	861 (67.1)	4807 (63.7)	0.020
Have not	3158 (35.8)	422 (32.9)	2736 (36.3)	
Physical activity ^a^				
Low	859 (9.7)	143 (11.1)	716 (9.5)	0.063
Moderate	2362 (26.8)	364 (28.4)	1998 (26.5)	
High	1177 (13.3)	154 (12.0)	1023 (13.6)	
Self-rated health status				
Good	2377 (26.9)	193 (15.0)	2184 (29.0)	< 0.001
Fair	4509 (51.2)	641 (50.0)	3868 (51.3)	
Poor	1940 (22.0)	449 (35.0)	1491 (19.7)	
Meteorological factor				
Ambient temperature (℃)	14.77 ± 4.73	14.58 ± 4.93	14.80 ± 4.69	0.118
Ambient RH (%)	71.30 ± 9.04	70.60 ± 9.24	71.41 ± 9.00	0.004

^a^ indicated physical activity is in the sub-sample. ^#^ the sum of the ratio is not 100% due to missing data

The mean exposure concentrations of PM_2.5_, PM_2.5−10_, NO_2_, SO_2_, CO, and O_3_ during the cross-sectional period were 66.13 (SD:22.68) µg/m^3^, 47.21 (20.97) µg/m^3^, 35.44 (10.84) µg/m^3^, 34.46 (17.02) µg/m^3^, 1.13 (0.39) mg/m^3^, 82.04 (13.63) µg/m^3^, respectively. Table 2 also shows the correlation between any two of the pollutants.

**Table 2:** Distribution and correlation of the exposure concentration of air pollutants

Air pollutants (µg/m^3^)	Mean	SD	Percentiles					Correlation					
			*P*_5_	*P*_25_	*P*_50_	*P*_75_	*P*_95_	PM_2.5_	PM_2.5−10_	NO_2_	SO_2_	CO	O_3_
PM_2.5_	66.13	22.68	31.75	48.70	66.85	79.10	104.80	1.00					
PM_2.5−10_	47.21	20.97	23.15	31.60	41.15	58.70	81.55	0.782^**^	1.00				
NO_2_	35.44	10.84	18.99	27.47	34.79	41.74	54.76	0.715^**^	0.570^**^	1.00			
SO_2_	34.46	17.02	13.48	23.43	28.82	46.24	66.31	0.697^**^	0.759^**^	0.522^**^	1.00		
CO	1.13	0.39	0.66	0.89	1.01	1.37	1.88	0.544^**^	0.555^**^	0.417^**^	0.574^**^	1.00	
O_3_	82.04	13.63	58.40	72.44	81.05	93.38	103.83	0.271^**^	0.354^**^	0.254^**^	0.244^**^	0.058^**^	1.00

Note: ^**^P < 0.01

**Fig. 1:**
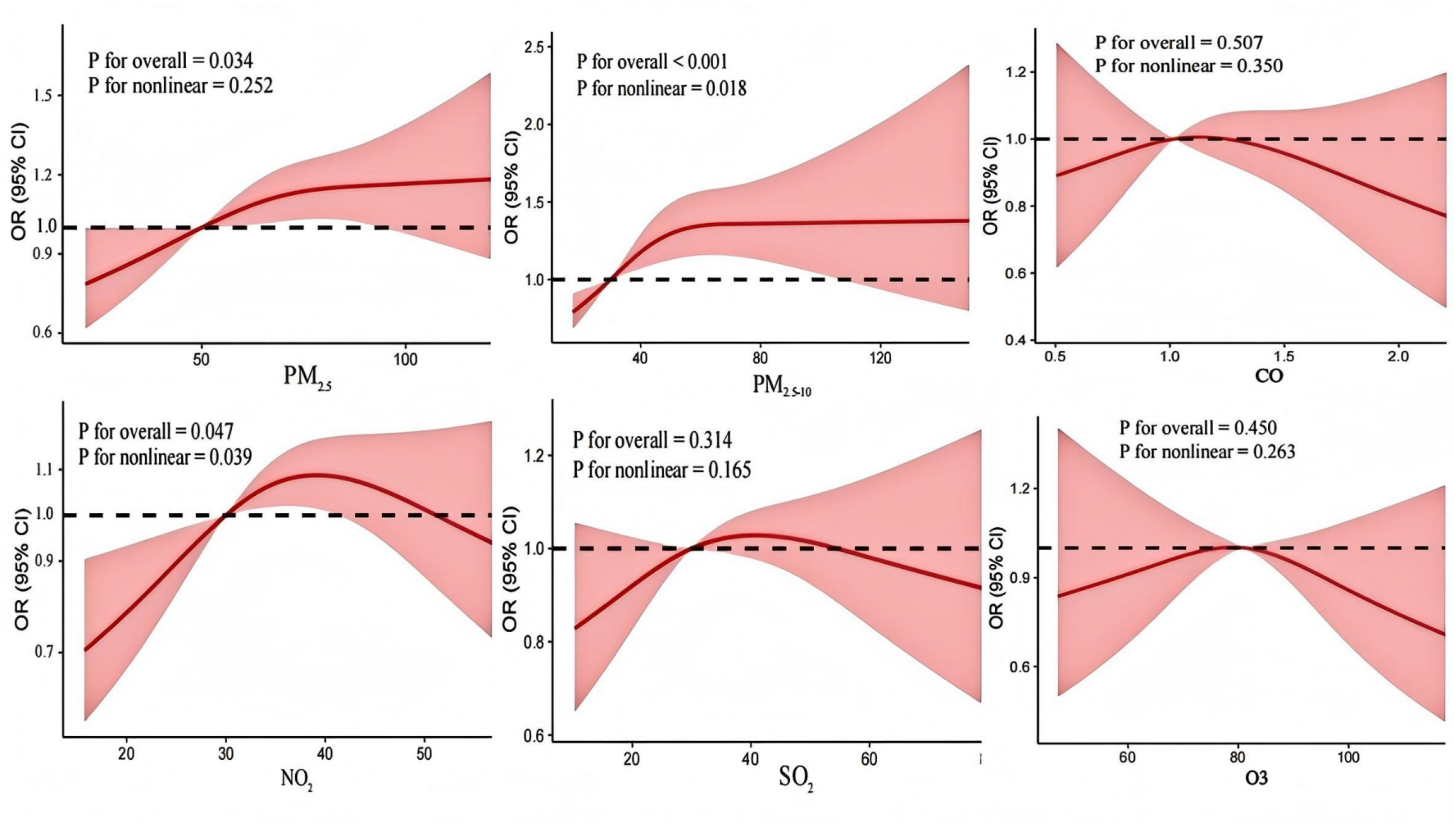
Exposure-response relationship between long-term air pollutants exposure with prevalent BPH. Note: model adjusted for age, BMI, education attainment, marital status, smoke, alcohol drink, self-rated health status, residence, ambient temperature, and relative humidity

Table 3 displays the cross-sectional associations of exposure to air pollutants with prevalent BPH. After controlling for confounders, each 10 µg/m^3^ increase in PM_2.5_ (OR 1.04, 95% CI: 1.01–1.07) and PM_2.5−10_ (1.06, 1.02–1.10) were positively associated with prevalent BPH. In the quartile analysis, the highest quartile levels of PM_2.5_ and PM_2.5−10_ were related to a higher prevalence of BPH, compared to the lowest quartile (P_trend_ <0.05). However, the quartile analysis showed no significance for NO_2_, SO_2_, CO, and O_3_ in the adjusted model (Table 3). We observed significant nonlinear relationships between PM_2.5−10_ and NO_2_ exposure with prevalent BPH (P_nonlinear_ <0.05, Fig. 1). Notably, the RCS curve for the association of PM_2.5_ and prevalent BPH is approximately linear overall (Fig. 1).

**Table 3:** Cross-sectional associations of long-term exposure to air pollutants with prevalent BPH

Air pollutants µg/m^3^	Model 1		Model 2	
	OR (95% CI)	*P*-value	OR (95% CI)	*P*-value
PM_2.5_				
Per 10 µg/m^3^	1.05 (1.03–1.08)	0.001	1.04 (1.01–1.07)	0.018
Q1	1.00	—	1.00	—
Q2	1.22 (1.02–1.45)	0.030	1.14 (0.92–1.41)	0.231
Q3	1.43 (1.21–1.70)	0.000	1.30 (1.05–1.60)	0.017
Q4	1.37 (1.15–1.63)	0.000	1.26 (1.02–1.56)	0.034
P for trend	—	0.000	—	0.018
PM_2.5−10_				
Per 10 µg/m^3^	1.07 (1.04–1.10)	0.000	1.06 (1.02–1.10)	0.002
Q1	1.00	—	1.00	—
Q2	1.64 (1.37–1.96)	0.000	1.63 (1.31–2.04)	0.001
Q3	1.49 (1.25–1.78)	0.000	1.31 (1.05–1.63)	0.019
Q4	1.68 (1.41–2.01)	0.000	1.59 (1.27–1.99)	0.001
P for trend	—	0.000	—	0.001
NO_2_				
Per 10 µg/m^3^	1.08 (1.02–1.14)	0.009	1.05 (0.98–1.12)	0.160
Q1	1.00	—	1.00	—
Q2	1.17 (0.99–1.40)	0.071	1.09 (0.88–1.34)	0.449
Q3	1.29 (1.09–1.53)	0.003	1.16 (0.94–1.42)	0.171
Q4	1.24 (1.05–1.47)	0.014	1.17 (0.95–1.44)	0.146
P for trend	—	0.007	—	0.121
SO_2_				
Per 10 µg/m^3^	1.03 (0.99–1.06)	0.145	1.01 (0.96–1.05)	0.821
Q1	1.00	—	1.00	—
Q2	1.17 (0.98–1.38)	0.078	1.12 (0.91–1.38)	0.269
Q3	1.22 (1.02–1.45)	0.026	1.07 (0.86–1.32)	0.547
Q4	1.22 (1.02–1.44)	0.025	1.07 (0.87–1.33)	0.512
P for trend	—	0.026	—	0.635
CO				
Per 1 mg/m^3^	1.05 (0.90–1.22)	0.529	0.99 (0.82–1.19)	0.877
Q1	1.00	—	1.00	—
Q2	1.01 (0.85–1.20)	0.905	0.95 (0.77–1.18)	0.649
Q3	1.09 (0.92–1.29)	0.312	1.02 (0.83–1.26)	0.823
Q4	1.21 (1.02–1.43)	0.027	1.10 (0.89–1.35)	0.378
P for trend	—	0.015	—	0.283
O_3_				
Per 10 µg/m^3^	1.01 (0.97–1.05)	0.711	0.98 (0.93–1.04)	0.556
Q1	1.00	—	1.00	—
Q2	1.37 (1.15–1.62)	0.001	1.17 (0.95–1.45)	0.136
Q3	1.24 (1.04–1.47)	0.014	1.16 (0.93–1.43)	0.183
Q4	1.16 (0.97–1.39)	0.108	1.07 (0.86–1.33)	0.560
P for trend	—	0.291	—	0.668

Model 1: crude model

Model 2: adjusted for age, BMI, education attainment, marital status, smoke, alcohol drink, self-rated health status, residence, ambient temperature, and relative humidity

**Fig. 2:**
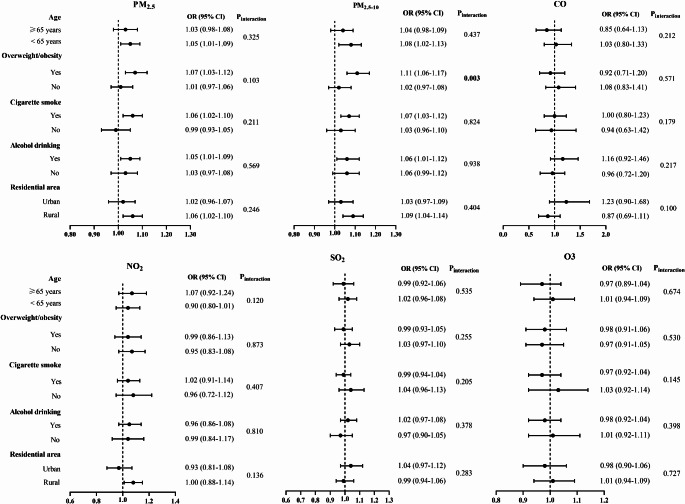
Association of air pollutants exposure with BPH stratified by age, BMI, residence, and behaviour characteristics. Note: the estimates were obtained from each 10 µg/m^3^ increment in air pollutants except for CO (1 mg/m^3^). Model adjusted for age, BMI, education attainment, marital status, tobacco smoke, alcohol use, self-rated health status, residence, ambient temperature, and relative humidity except for the stratified variable

Figure 2 shows the subgroup analysis of the association with BPH by age, BMI, place of residence, and behavior characteristics separately. We observed a higher risk of BPH in individuals who were overweight/obesity after exposure to PM_2.5−10_ (*p* interaction = 0.003). However, the association with BPH was not modified by age, smoke, alcohol drinking, and residence (Fig. 2).

After further adjusting for other air pollutants, the two-pollutant models showed similar results for PM_2.5_ and PM_2.5−10_ (Table S1). Sensitivity analysis by using five-year air pollutant exposure showed a robust association of PM_2.5_ and PM_2.5−10_ with BPH (Table S2). By considering physical activity in the sub-sample, a significant association was observed for PM_2.5−10_ (Table S3).

## Discussion

Our study provided additional evidence to investigate the associations of multiple air pollutants with BPH risk. Long-term exposure to PM_2.5_ and PM_2.5−10_ were associated with a higher prevalence of BPH among middle-aged and older Chinese men. There were non-linear relationship between PM_2.5−10_ and NO_2_ with BPH. The association was more evident in individuals who were overweight/obesity after exposure to PM_2.5−10_. Sensitivity analyses proved the stability of our findings.

The prevalence of BPH was 14.5% among the participants, close to that in a previous study (13.1%) in China (Zhang et al., 2022b). Currently, only one study by *Shim et al.* examined the association of air pollutants with BPH in Korea, and they found PM exposure was in relation to BPH (Shim et al. 2016), in line with our findings. However, Korea’s study (Shim et al. 2016) assessed the associations using an ecological study, with a relatively small sample size (*n* = 1734), and might have more confounding. By contrast, our national study added the knowledge by analyzing the associations of air pollutant exposure with BPH in China. The findings of our study are the first to reveal an association between PM_2.5−10_ with BPH in China. The sources of PM_2.5−10_ are from windblown dust, traffic vehicles, and agriculture activities, whereas PM_2.5_ is primarily from the combustion processes (Peng et al. 2008). An in-vivo study has shown that PM_2.5−10_ exposure is more likely to induce extensive interstitial inflammations compared to PM_2.5_ (Happo et al. 2010), which is related to the development of BPH. Nonetheless, future investigations are warranted to verify our findings. In addition, the RCS curve demonstrated a significant exposure-response relationship between NO_2_ with prevalent BPH, echoing the positive association found in Korea’s study (Shim et al. 2016).

To date, the mechanisms underline the associations of air pollution with BPH remain unclear, while some possible explanations have been favored. One explanation is that PM can directly impact the prostate by disrupting cell membranes and causing apoptosis via mitochondrial dysfunction (Youogo et al. 2022). Furthermore, it is suggested that the major source of PM_2.5−10_ was industrial and traffic emissions in China (Chen et al. 2019). The chemical components of PM, i.e., polycyclic aromatic hydrocarbons (PAH_S_), benzene, and metal may act as endocrine disruptors and adversely affect prostate health, leading to the progress of BPH (Radwan et al. 2016; Shim et al. 2016). Compared to PM_2.5_, PM_2.5−10_ is more likely to deposit in the upper and larger airways, thereby increasing the risk of asthma (Peng et al. 2008). It is suggested that asthma is related to the risk of BPH due to chronic inflammation (Peng et al. 2020). In addition, epidemiological evidence has linked higher PM exposure to elevated testosterone levels in men (Wei et al. 2021a; Zhou et al. 2018). Given that the progression of BPH is believed to depend on high androgen levels (Rastrelli et al. 2019), this may partially explain the mechanisms.

Our stratification analysis suggested that individuals who were overweight/obesity had a higher risk of BPH after PM_2.5−10_ exposure. A previous Mendelian randomization (MR) study showed an independent role of high BMI with an increased risk of BPH (Wang et al. 2022). Moreover, evidence indicated obesity could drive a state of chronic inflammation and oxidative stress, resulting in prostate tissue immune cell infiltration, tissue remodeling, and clinical BPH (Furukawa et al. 2004; Parsons et al. 2013).

Our study has several notable strengths. First, it was the first national study to explore the associations of various air pollutants with BPH risk among middle-aged and older men in China. The findings contributed to adding the limited evidence on outdoor PM_2.5_ and PM_2.5−10_ with BPH in men. Second, the nationally representative sample coupled with the high-resolution satellite models helps improve the quality of findings. Furthermore, the robust association of PM_2.5−10_ with prevalent BPH, which, for the first time, highlights the development of public health regulations of coarse PM to prevent BPH in China.

Some limitations should be acknowledged. First, due to data limitations in the CHARLS, we could only estimate air pollutant concentrations at the county-city level, However, the measurement error generally biases toward the null (Hutcheon et al. 2010). Second, the CHARLS questionnaire did not allow us to assess BPH and its severity using established clinical tests. While prior studies have suggested that the CHARLS question for diagnosing BPH was reliable (Xiong et al. 2021; Zhang et al. 2019), the potential misclassification might exist. Hence, caution should be paid to these findings. Moreover, the LUTS could not be evaluated due to the absence of a definite diagnosis in the CHARLS. Last, the unobserved and residual confounding such as diets and hormone status could not be considered due to unavailable data.

## Conclusions

This study firstly demonstrated that long-term PM_2.5_ and PM_2.5−10_ exposure were associated with prevalent BPH among middle-aged and older men in China. The association was more pronounced in individuals who were overweight/obesity. The findings highlight that environmental interventions should be tailored to improve prostate health in men by reducing air pollution.

## Electronic supplementary material

Below is the link to the electronic supplementary material.

Supplementary Material 1

## Data Availability

This study used the open-access dataset which can be applied from the website http://charls.pku.edu.cn/en/.
