# Supplementary material for: Association of long-term exposure to air pollutants with benign prostatic hyperplasia among middle-aged and older men in China
Source: Int Arch Occup Environ Health. 2025 Mar 14;98(3):321–9. doi: 10.1007/s00420-025-02127-w (PMC11972197; doi:10.1007/s00420-025-02127-w)
Supplement: Supplementary file 1 — Supplementary Material 1 [file 420_2025_2127_MOESM1_ESM.doc]

**Supporting information**

**Association of long-term exposure to air pollutants with benign prostatic hyperplasia among middle-aged and older men in China**

**Contents**

eMethods

Table S1. Sensitivity analyses of air pollutants exposure with BPH in the two-pollutant model.

Table S2. Sensitivity analyses of five-year average air pollutants exposure with prevalent BPH.

Table S3. Sensitivity analysis of air pollutants with BPH by further adjusting for physical activity in the sub-sample.

Figure S1. Map of the 125 study cities.

Figure S2. Selection process of the study participants.

Figure S3. Directed Acyclic Graph for the association of air pollution exposure with BPH.

***eMethods***

The CHARLS used a multistage probability sampling strategy to select respondents (Zhao et al., 2014). First, the district and county units were stratified by region, urban or rural areas, and GDP per capital. Using the Probability Proportional to population Size (PPS) method, 150 district and county units were selected randomly, and within each unit, three villages or community units (psus) were chosen (Zhao et al., 2014; Gong et al., 2022). The CHARLS team developed a special mapping software to create a sample frame of all households in each psu. One resident aged 45 years and older was randomly selected as the main respondent from each household, and their spouse was also included in the survey (Zhao et al., 2014).

In the current study, a total of 8,826 individuals from 125 county-cities (min-max size: 40,674 to 2,091,869 county population) in 28 provinces were screened for the final analyses.

**Table S1. Sensitivity analyses of air pollutants exposure with BPH in the two-pollutant model.**

| Air pollutants | Adjusted model | |
| --- | --- | --- |
|  | aOR (95% CI) | P-value |
| PM_2.5_ |  |  |
| +PM_2.5-10_ | 1.00 (0.95-1.05) | 0.974 |
| +NO_2_ | 1.05 (1.00-1.10) | **0.049** |
| +SO_2_ | 1.07 (1.02-1.12) | **0.003** |
| +CO | 1.06 (1.02-1.10) | **0.003** |
| +O_3_ | 1.04 (1.01-1.08) | **0.011** |
| PM_2.5-10_ |  |  |
| +PM_2.5_ | 1.06 (1.00-1.12) | **0.049** |
| +NO_2_ | 1.06 (1.02-1.11) | **0.006** |
| +SO_2_ | 1.09 (1.04-1.14) | **0.001** |
| +CO | 1.08 (1.04-1.13) | **0.001** |
| +O_3_ | 1.07 (1.03-1.10) | **0.001** |
| NO_2_ |  |  |
| +PM_2.5_ | 0.97 (0.88-1.08) | 0.609 |
| +PM_2.5-10_ | 0.99 (0.91-1.07) | 0.716 |
| +SO_2_ | 1.06 (0.98-1.15) | 0.128 |
| +CO | 1.06 (0.99-1.14) | 0.107 |
| +O_3_ | 1.06 (0.99-1.13) | 0.119 |
| SO_2_ |  |  |
| +PM_2.5_ | 0.95 (0.89-1.01) | 0.066 |
| +PM_2.5-10_ | 0.94 (0.88-1.00) | 0.052 |
| +NO_2_ | 0.98 (0.93-1.04) | 0.536 |
| +CO | 1.01 (0.96-1.06) | 0.714 |
| +O_3_ | 1.01 (0.96-1.05) | 0.733 |
| CO |  |  |
| +PM_2.5_ | 0.81 (0.65-1.02) | 0.079 |
| +PM_2.5-10_ | 0.82 (0.66-1.01) | 0.070 |
| +NO_2_ | 0.92 (0.74-1.13) | 0.424 |
| +SO_2_ | 0.96 (0.77-1.21) | 0.745 |
| +O_3_ | 0.99 (0.82-1.19) | 0.887 |
| O_3_ |  |  |
| +PM_2.5_ | 0.97 (0.92-1.02) | 0.265 |
| +PM_2.5-10_ | 0.96 (0.91-1.01) | 0.159 |
| +NO_2_ | 0.98 (0.92-1.03) | 0.371 |
| +SO_2_ | 0.98 (0.93-1.04) | 0.521 |
| +CO | 0.98 (0.92-1.04) | 0.558 |

Model adjusted for age, BMI, educational attainment, marital status, smoking status, alcohol consumption, self-rated health status, residence, ambient average temperature, and relative humidity

**Table S2. Sensitivity analyses of five-year average air pollutants exposure with prevalent BPH.**

| Air pollutants  μg/m^3^ | Sensitivity analysis | | Main analysis | |
| --- | --- | --- | --- | --- |
|  | aOR (95% CI) | P-value | aOR (95% CI) | P-value |
| PM_2.5_ | 1.05 (1.01-1.08) | 0.010 | 1.04 (1.01-1.07) | 0.018 |
| PM_2.5-10_ | 1.07 (1.03-1.11) | 0.001 | 1.06 (1.02-1.10) | 0.002 |
| NO_2_ | 1.02 (0.99-1.06) | 0.245 | 1.05 (0.98-1.12) | 0.160 |
| O_3_ | 1.02 (0.97-1.08) | 0.158 | 0.98 (0.93-1.04) | 0.556 |

Note: the estimates were calculated from each 10 μg/m^3^ increment in air pollutants. The estimates cannot be calculated due to unavailable data for SO_2_ and CO in the CHAP before 2013.

Model adjusted for age, BMI, education attainment, marital status, smoke, alcohol drink, self-rated health status, residence, ambient temperature, and relative humidity.

**Table S3. Sensitivity analysis of air pollutants with BPH by further adjusting for physical activity in the sub-sample.**

| Pollutants | Model 1 | | Model 2 | |
| --- | --- | --- | --- | --- |
|  | OR (95% CI) | P-value | OR (95% CI) | P-value |
| PM_2.5_ | 1.05 (1.01-1.09) | 0.009 | 1.03 (0.98-1.07) | 0.224 |
| PM_2.5-10_ | 1.08 (1.04-1.12) | 0.001 | 1.06 (1.01-1.12) | 0.026 |
| NO_2_ | 1.09 (1.01-1.17) | 0.028 | 1.05 (0.95-1.15) | 0.352 |
| SO_2_ | 1.06 (1.01-1.11) | 0.020 | 1.01 (0.95-1.07) | 0.780 |
| CO | 1.06 (0.86-1.31) | 0.573 | 0.91 (0.70-1.18) | 0.479 |
| O_3_ | 1.01 (0.95-1.08) | 0.676 | 0.98 (0.91-1.05) | 0.548 |

Model 1: crude model;

Model 2: adjusted for age, BMI, educational attainment, marital status, smoking status, alcohol consumption, self-rated health status, residence, ambient average temperature, relative humidity, and physical activity.


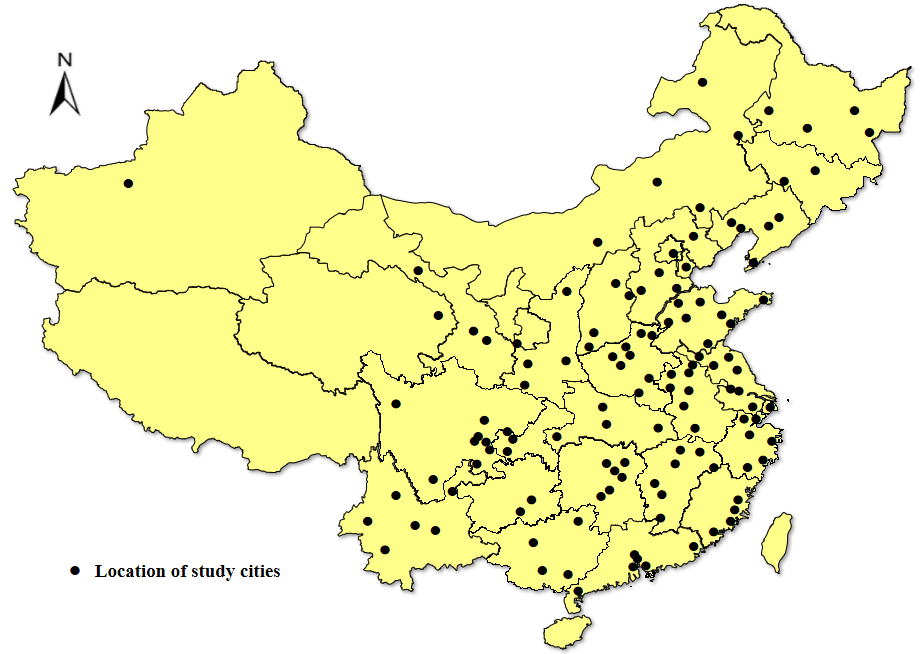


**Figure S1. Map of the 125 study cities.**


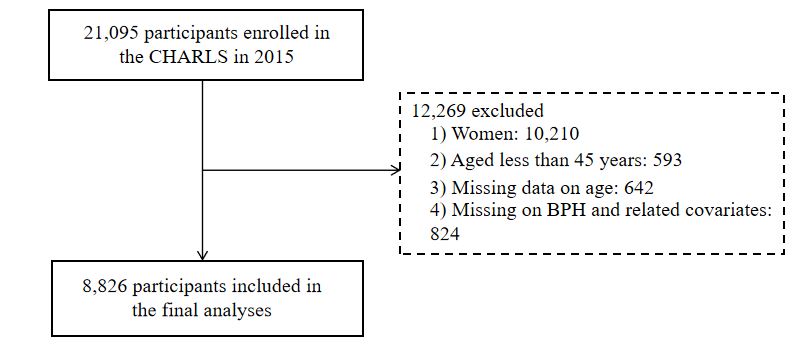


**Figure S2. Selection process of the study participants.**


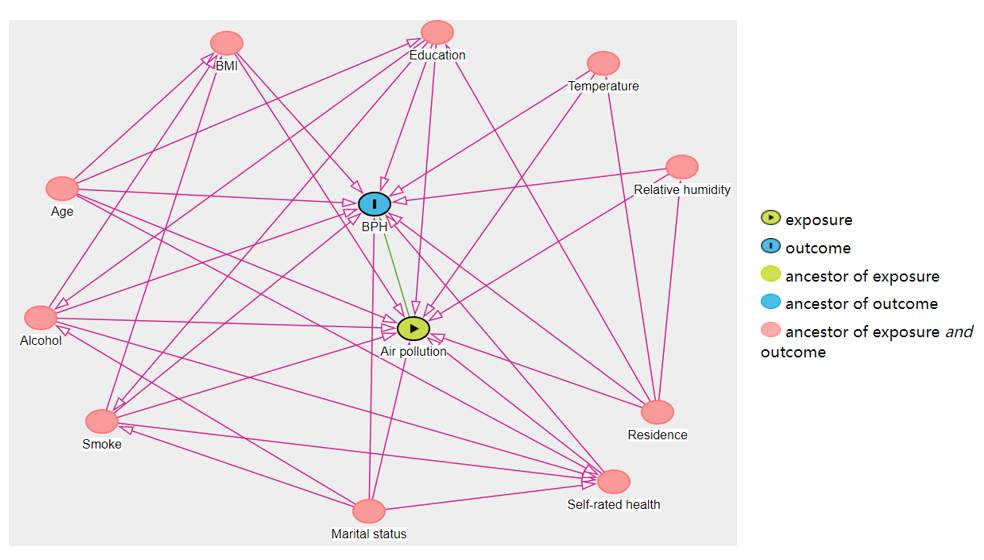


**Figure S3. Directed Acyclic Graph for the association of air pollution exposure with BPH.**

**References**

Gong, J., Wang, G., Wang, Y., Chen, X., Chen, Y., and Meng, Q. et al. (2022) Nowcasting and forecasting the care needs of the older population in China: analysis of data from the China Health and Retirement Longitudinal Study (CHARLS). *Lancet Public Health* **7**: e1005-e1013.

Zhao, Y., Hu, Y., Smith, J.P., Strauss, J., and Yang, G. (2014) Cohort profile: the China Health and Retirement Longitudinal Study (CHARLS). *Int J Epidemiol* **43**: 61-68.
